# A Single Strain of *Lactobacillus* (CGMCC 21661) Exhibits Stable Glucose- and Lipid-Lowering Effects by Regulating Gut Microbiota

**DOI:** 10.3390/nu15030670

**Published:** 2023-01-28

**Authors:** Yuying Wang, Xiaozhong Wang, Xinzhu Xiao, Shufang Yu, Wennan Huang, Benqiang Rao, Fenglin Chen

**Affiliations:** 1 Department of Gastrointestinal Surgery, Capital Medical University Affiliated Beijing Shijitan Hospital, Beijing 100038, China; 2 Department of Gastroenterology, Fujian Medical University Union Hospital, Fuzhou 350001, China

**Keywords:** type 2 diabetes, *Lactobacillus*, microbial transplantation, anti-inflammatory, glucose metabolism, lipid metabolism

## Abstract

Type 2 diabetes (T2D) is usually accompanied by obesity and nonalcoholic fatty-liver-related insulin resistance. The link between T2D and dysbiosis has been receiving increasing attention. Probiotics can improve insulin sensitivity by regulating imbalances in microbiota, but efficacy varies based on the probiotic used. This study screened the main strain in the feces of healthy adult mice and found it to be a new *Lactobacillus* (abbreviated as *Lb.*, named as CGMCC No. 21661) after genetic testing. We designed the most common *Bifidobacterium longum subsp. longum* (CGMCC1.2186, abbreviated as *B. longum. subsp.*), fecal microbiota transplantation (FMT), and *Lb.* CGMCC No. 21661 protocols to explore the best way for modulating dysbiosis to improve T2D. After 6 weeks of gavage in T2D mice, it was found that all three protocols had a therapeutic alleviating effect. Among them, compared with the *B. longum. subsp.* and FMT, the *Lb.* CGMCC No. 21661 showed a 1- to 2-fold decrease in blood glucose (11.84 ± 1.29 mmol/L, *p* < 0.05), the lowest HOMA-IR (*p* < 0.05), a 1 fold increase in serum glucagon-like peptide-1 (5.84 ± 1.1 pmol/L, *p* < 0.05), and lowest blood lipids (total cholesterol, 2.21 ± 0.68 mmol/L, *p* < 0.01; triglycerides, 0.4 ± 0.15 mmol/L, *p* < 0.01; Low-density lipoprotein cholesterol, 0.53 ± 0.16 mmol/L, *p* < 0.01). In addition, tissue staining in the *Lb.* CGMCC No. 21661 showed a 2- to 3-fold reduction in T2D-induced fatty liver (*p* < 0.0001), a 1- to 2-fold decrease in pancreatic apoptotic cells (*p* < 0.05), and a significant increase in colonic mucus layer thickness (*p* < 0.05) compared with the *B. longum. subsp.* and FMT. The glucose and lipid lowering effects of this *Lb.* CGMCC No. 21661 indicate that it may provide new ideas for the treatment of diabetes.

## 1. Introduction

Diabetes is the third-largest chronic non-communicable disease in the world. With the progress of social aging and urbanization, according to the International Diabetes Federation data, an estimated 463 million people suffered from diabetes in 2019, accounting for 9.3% of the global population [1]. This number is expected to rise to 700 million by 2045. Type 2 diabetes (T2D) accounts for more than 90% of the total prevalence and poses a significant risk to human health [2]. T2D is attributable to a range of factors including nutrition, physical exercise, impaired glucose tolerance, abnormal lipid metabolism, and disorders of the intestinal micro-ecosystem [3]. Intestinal microbes and their metabolites work with host cells to maintain normal physiological functions, such as fatty acid metabolism, bile acid metabolism, and maintenance of the intestinal barrier, all of which affect the occurrence and development of T2D [4,5,6]. The intestinal barrier system is a habitat for microorganisms, and dysfunctional composition and function of the intestinal microbiota changes the permeability of the intestinal lumen. The microbiota refers to the complete system of microorganisms in the gut. An intact intestinal barrier has ability to absorb nutrients, induces immune response, and restricts bacteria entering into the blood. Gram-negative bacterial cell wall component lipopolysaccharide (LPS), increases the release of inflammatory factors through the toll-like receptor 4-mediated inflammatory pathway, causing chronic low-grade inflammation [7]. Furthermore, inflammation has a cascading effect on host obesity and insulin resistance (IR). Probiotics can compete with harmful bacteria for nutrition and adhesion sites and produce inhibitory compounds (such as bacteriocins), which alter the composition of gut microbiota. The simultaneous production of short-chain fatty acids by these probiotics reduces the pH of the colon, increases the release of the intestinal hormone glucagon-like peptide-1 (GLP-1), and improves insulin sensitivity [8].

In the past two decades, treatment for dysbiosis in T2D has received increasing attention. Human and animal studies have shown that modulating the gut microbiota through probiotics, prebiotics, synbiotics, or fecal microbial transplants (FMT) has a positive effect on lowering blood glucose and lipids and improving T2D [9,10,11,12]. Therefore, the use of probiotics for the treatment of metabolic diseases has become a popular topic in gut microbiome research. At present, there is sufficient evidence that dysbiosis is closely related to T2D, but the quality and efficacy of the studies, and the selection of strains, are highly controversial [13,14,15]. *Lactobacillus* (abbreviated as *Lb.*) and *Bifidobacterium longum* (abbreviated as *B. longum.*) are common probiotics, and they both have cholesterol- and glucose-lowering effects [8,16]. *Lb.* has previously been found to have a range of positive impacts on high-fat diet (HFD)-induced obese mice. It improves T2D by regulating dysbiosis and has similar hypoglycemic effects to metformin, while reducing intestinal inflammation and increasing GLP-1 secretion [17,18]. However, different studies with different selection of *Lb.* produced different effects [8,19,20]. Another bacterial strain, *B. longum.*, has been widely used in commercial probiotics and has been reported to protect the host from enteropathogenic infection through the production of acetate [21]. There are many reports of digesting food [22], breaking down lactose [23], and colonizing the gut. It has also been found to reduce the levels of inflammatory cytokines by producing butyric acid, which improves IR [24]. In some human studies, *B. longum.* were found to inhibit Dipeptidyl Peptidase-4 (DDP-4), but not increase insulin sensitivity [25,26]. Acarbose, an α-glucosidase inhibitor, further enhances the hypoglycemic effect of antidiabetic drugs by effectively increasing the number of probiotics to regulate dysbiosis, while probiotics can also lower blood glucose by enhancing α-glucosidase inhibition [27,28]. Therefore, some studies have combined probiotics, such as *Lb.* or *B. longum.,* with acarbose for antidiabetic treatment; however, these studies may have overlooked the role of single strains to some extent [29]. In addition, FMT can relieve symptoms associated with T2D [30]. In multiple previous studies, feces from lean mice were transplanted into obese mice via FMT, and it was found that certain metabolic processes were altered and the mice lost weight [31]. However, this approach is also imperfect as it caused a wide range of adverse clinical reactions [32]. In addition, it has been difficult to apply on a large scale due to ethical concerns and allograft rejection, and metformin is still needed.

*Bifidobacterium longum subsp. longum*, CGMCC1.2186 (JCM1217) (abbreviated as *B. longum. subsp.*) is often used as a probiotic control group in research [33]. In addition, FMT can relieve symptoms associated with T2D [30]. Therefore, we selected the control groups that are most likely to alleviate the symptoms of T2D mice and improve the composition of intestinal microbiota, in order to explore the best solution for T2D improvement. In order to find an effective strategy for a single probiotic strain to treat T2D by improving dysbiosis, we screened probiotic strains of *Lb.* CGMCC No. 21661 from mouse feces for their therapeutic effects on T2D. In this study, a strain of *Lb.* CGMCC No. 21661 was screened for intervention with *B. longum. subsp.* and FMT, respectively, to explore the best solution for improving T2D. Our study found that the screened *Lb.* CGMCC No. 21661 were most effective in lowering glucose and lipids, which is expected to provide a new idea for the treatment of T2D.

## 2. Materials and Methods

### 2.1. High-Fat Diet/Streptozotocin-Induced Diabetic Mice

Fifty-five 6-week-old male C57BL/6J mice were randomly divided into normal feed control group (control, *n* = 10) and T2D model group (*n* = 45). The model group was fed an HFD for 4 weeks, and 40 mg/kg low-dose streptozotocin (HFD/STZ) was injected intraperitoneally for 5 days [34]. After another week of feeding with HFD, the fasting blood glucose of the mice was greater than 11.1 mmol/L. Mice in the control group had a normal diet and free drinking water.

### 2.2. Experimental Conditions

Animals were housed at the Experimental Animal Center of Fujian Medical University (Fuzhou, China). The experiment started after 1 week of normal diet feeding. During the experiment, five mice were kept in a cage at 22 ± 1 ℃, 55 ± 5% humidity, 12 h of light and dark automatic cycle, and were allowed to freely eat and drink (HFD: D12492, 60 kcal% fat, Research Diets, lnc. (New Brunswick, NJ, USA); normal diet: 4.9 kcal% fat, (Table A1)). We placed molar rods in each cage to prevent the HFD food from hardening and affecting the eating patterns of the mice. After modeling, we added two bottles of sterilized water to each cage to ensure sufficient drinking water. Bedding was changed every 24 h, and the diet and activity of the mice were observed.

We purchased C57BL/6J mice (5-week-old, male, 15 ± 0.7 g) from Beijing Huafukang Biotechnology Co., Ltd. (Beijing, China). The animal study was reviewed and approved by Institutional Animal Care and Use Committee (IACUC) of the Fujian Medical University following the Guidelines for the Care and Use of Laboratory Animals (approval number: FJMU IACUC 2020-0019).

### 2.3. Bacterial Culture Conditions and Administration to Mice

Anaerobic bacteria make up the majority of intestinal microbiota and play important functions in transporting and decomposing glucose. Therefore, we used an anaerobic environment and modified-de Man, Rogosa and Sharpe (MRS) medium (each 100 mL modified-MRS medium plus 0.05 g L-cysteine hydrochloride, 5 mg mupirocin lithium salt and 0.6 g agar powder) to screen for dominant strains [35]. We needed 10 mL of MRS medium to make 1 solid medium. We collected feces from adult mice (10 weeks old) into tubes containing phosphate-buffered saline (PBS) solution (1 mL of 0.1 mM). Then we took 100 mL of the supernatant and cultured it in modified-MRS medium. After culturing at 37 °C for 72 h, we selected a single colony and then re-cultured it. Large-diameter colonies were cloned and stored separately. We used freshly cultured bacterial liquid to gavage the mice in the fecal dominant microbiota (FDM) group. After the experiment, the FDM was sequenced and identified using Illumina HiSeq™ sequencing (Sangon Biotech, Inc., Shanghai, China).

We purchased *B. longum. subsp.* from China General Microbiological Culture Collection Center (CGMCC) (Beijing, China), and the strain name was *Bifidobacterium longum subsp*. *longum*, CGMCC1.2186 (JCM1217). This strain was used to study the effect of *B. longum.* in the treatment of diabetes.

We collected fresh fecal samples from the control group mice (10–12 weeks) between 9–10 a.m., after which the samples were placed in sterile cryogenic vials, quick-frozen in liquid nitrogen, and stored at −80 °C. Before transplantation, samples were pooled and immediately homogenized using ice-cold PBS (120 mg feces/1 mL) followed by centrifugation at 800 g/3 min/4 °C. The supernatant was used for FMT group.

### 2.4. Bacterial Sequencing and Annotation

We collected feces from post-intervention mice for 16s sequences [36]. Bacterial DNA was extracted from mouse feces. We took 200 mg mouse fecal samples and left the samples after shaking with 1 mL PBS solution. DNA was extracted with E.Z.N.A^TM^ Mag-Bind Soil DNA Kit (Omega Bio-Tek, Norcross, GA, USA), and the integrity of DNA was checked with agarose gel electrophoresis. Genomic DNA was accurately quantified using the Qubit 3.0 DNA Assay Kit (Omega Bio-Tek, Norcross, Norcross, GA, USA) to determine the amount of DNA that should be added to the PCR reaction. The hypervariable V3–V4 region of the 16s gene was PCR with barcoded 341F (5′-3′) and reverse primer 805R (5′-GACTACHVGGGTATCTAATCC-3′). PCR was performed in a thermocycler with a reaction volume of 25 μL and the following program settings: 95 °C for 3 min; 25 cycles: 95 °C for 30 s, 55 °C for 30 s, 72 °C for 30 s; final extension at 72 °C for 5 min; and maintained at 4 °C. The PCR products were purified and recovered using 0.6x magnetic beads, and then sequenced using Illumina HiSeq™ sequencing (Sangon Biotech, Inc., Shanghai, China). Then we performed operational taxonomic unit (OTU) clustering and taxonomy assignment, and analyzed using MEGAN, MG-RAST, and QIIME 4 analysis tools.

### 2.5. Microbiota Transplantation Treatment

Two mice died after modeling. The surviving T2D mice were randomly divided into four groups, PBS buffer (HFD-STZ, *n* = 10), fecal dominant microbiota (FDM, *n* = 9), *B. longum. subsp.* (BIO, *n* = 10), and fecal microbiota transplantation (FMT, *n* = 14) groups. We gavaged mice with 1.2 × 105 CFU/d of bacterial fluid for four weeks, once every 3 days in the 5th week, and once in the 6th week. During the experiment, we monitored fasting blood glucose, body weight and diet.

### 2.6. Serological Index Detection

After fasting overnight, the mice were immobilized, the tails were sterilized, and the tails were clipped about 0.5 cm to allow the natural flow of blood into tubes containing sodium citrate anticoagulant. They were then tested for hemoglobin A1c (HbA1c). After that, we collected retro-orbital blood into blood collection tubes after removing one eyeball of the mouse [37]. We separated retro-orbital blood into serum, and then detected total cholesterol (TC), triglyceride (TG), low-density lipoprotein-cholesterol (LDL-C), high-density lipoprotein-cholesterol (HDL-C), and the aspartate aminotransferase/alanine aminotransferase (AST/ALT) ratio [38]. We used the enzyme-linked aptamer sorbent assay (ELASA) kit to measure serum INS, GLP-1, DDP-4 and inflammatory factors (Interleukin-10 (IL-10), Tumour necrosis factor alpha (TNF-α)) [39,40,41]. For the test, specimens, standards and HRP-labeled antibodies are added to microtiter wells pre-covered with antibodies, followed by incubation, washing, and color development. The shade of color is positively correlated with the concentration of the specimen to be tested. The ELISA kit was purchased from Shanghai Youxuan Biotechnology Co., Ltd. (Shanghai, China).

### 2.7. Histological Analysis

The liver, pancreas, and colon tissues of the mice were either fixed with 4% paraformaldehyde for paraffin embedding or frozen. Hematoxylin and eosin (HE) staining of liver paraffin sections were used to observe structure and assessed using a three-stage scoring system: mild (score 1, 5–33%), moderate (score 2, 34–66%), and severe diffuse (score 3, 66%) [42,43]. Oil Red O staining of liver frozen sections were used to identify intracellular lipid droplets. TUNEL staining to observe islet apoptotic cells, HE staining to observe the quantity and quality of the islet cells, colon Alcian blue staining to observe the thickness of mucus layer [44,45]. Then we measured them by Image-Pro Plus 6.0 (MEDIA CYBERNETICS, Rockville, MD, USA).

### 2.8. Semi-Quantitative Western Blotting

We incubated 30 mg colon tissue in RIPA lysis buffer (50 mM Tris–HCl, pH 7.4; 150 mM NaCl, 1% Nonidet P-40) containing protease inhibitors (2 mM phenylmethylsulphonyl fluoride, NaVO_3_). The supernatant was then collected by centrifugation. After quantification by the Bicinchoninic acid (BCA) method, it was separated and electrophoresed on 10% SDS-PAGE, and then incubated with GLP-1 antibody, rabbit anti-mouse antibody. We performed semi-quantitative detection of the GLP-1 protein through western blotting [46].

### 2.9. Bacterial Genetic Analysis

We used National Center for Biotechnology Information (NCBI) BLAST+ to compare the 16s rRNA sequences from Illumina sequencing with the NCBI 16s database and obtained homology information. The analysis was set to identify sequences with >95% similarity. We selected the 30 16s rRNA sequences with the highest identity and used MEGA-X for multiple sequence alignment to construct a phylogenetic tree. By comparing with NCBI non-redundant protein sequences (NR), we were able to measure transcript similarity between species and obtain functional information about homologous sequences. We performed genetic and protein expression analysis of *Lb.* CGMCC 21661 [47,48]. We performed functional profiles of pathway enrichment analysis using the kyoto encyclopedia of genes and genomes (KEGG). Statistical genes fall into three categories: biological processes, cellular components, and molecular functions. KEGG integrates genomic, chemical, and systemic functional information for the systematic analysis of gene product functions and their intracellular metabolic pathways. Furthermore, we used BLAST to compare the gene protein sequences with the virulence factors of pathogenic bacteria (VFDB) database specialized in pathogenic bacteria and combined the functional annotation information of genes and their corresponding virulence factors to obtain the annotation results. We used BLAST to align the gene protein sequences with the comprehensive antibiotic resistance database (CARD) drug resistance gene database or pathogen host interactions database (PHI) based pathogen-host interaction database and combined the genes and their corresponding functions to obtain the annotation results.

### 2.10. Statistics

We used SPSS 25.0 for statistical analysis and GraphPad Prism 8.4 for drawing. Single-factor or multi-factor analysis of variance was used, and F-test for the analysis of variance. Data were expressed as mean ± standard (Mean ± SD) deviation, and *p* < 0.05 is considered statistically significant.

## 3. Results

### 3.1. Identifying Dominant Strains for Fecal Screening

**Figure 1:**
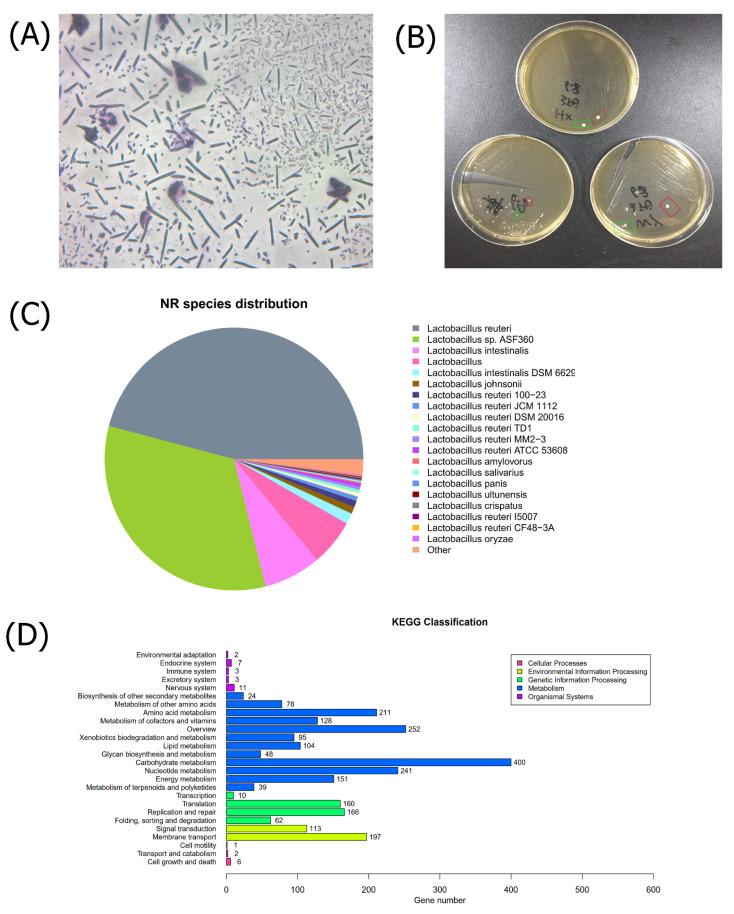
Identification of bacteria. (**A**) Gram staining is positive (200×). (**B**) The hydrogen peroxide test is negative. (**C**) Pie chart of homologous species classification in NR database, where each sector represents a species. The larger the fan-shaped area, the greater the number of similar sequences between the FDM strain and this strain. The area of fan-shaped is less than 50%. (**D**) Kyoto encyclopedia of genes and genomes (KEGG) for functional annotation of genomes or transcriptomes.

**Figure A1:**
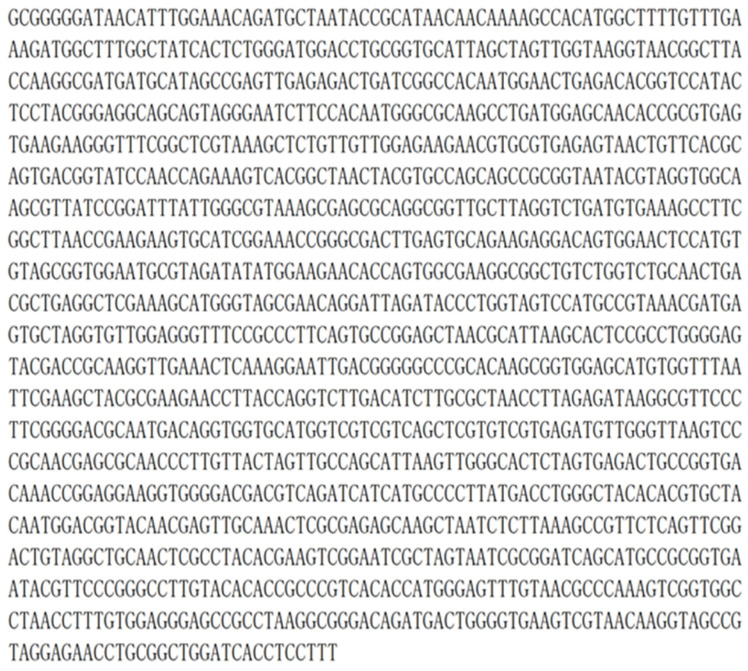
16s rRNA sequencing results.

The experiments verified that the dominant strains selected in this study have a significant role in treating T2D, lowering glucose levels, lowering lipids, and improving inflammation. Cultured strains from FDM were sequenced if they returned a positive gram stain and a negative peroxide test (Figure 1A,B). After using Illumina HiSeq™ sequencing, we used NCBI Blast+ to align16s rRNA sequences to FDM (Figure A1). The 30 16s rRNA sequences with the highest identity were not on the same branch, and the similarity was 61%. This indicates that the cultured strain belonged to the genus *Lb.* In the NR database for transcriptomic comparison (Figure 1C), the sequence similarity between the FDM strains and existing strains was less than 50%, indicating a species different from the existing strains. The new *Lb.* strain was named *Lb.* CGMCC 21661.

To more clearly characterize the *Lb.* CGMCC 21661, we performed genetic analysis. We comprehensively characterized bacteria at the protein, genetic, and functional levels using KEGG (Figure 1D), respectively. VFDB from various best-characterized bacterial pathogens, with emphasis on functional and structural biology, and immunology. VFDB results were divided into two groups, SetA and SetB (Table 1). The VFDB of the bacteria was about 6%, and there was no characteristic pathogenicity. A comparison of CARD and PHI showed that this bacterium has no definite pathogen to infect the host (Table 2 and Table 3).

### 3.2. Bacterial Transplantation Reduces T2D-Induced Hyperglycemia

**Figure 2:**
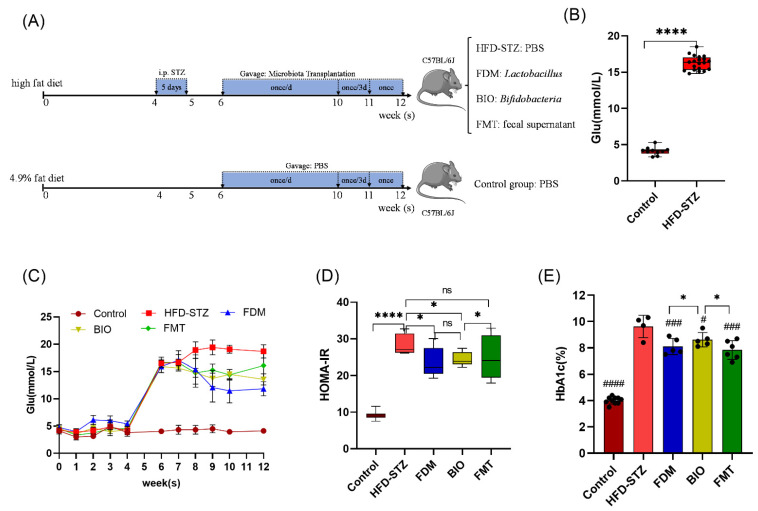
Glucose-lowering effect of bacterial transplantation into mice models of type 2 diabetes. (**A**) Flow chart of the model development. (**B**) Fasting blood glucose value of the mice after modeling; the blood glucose levels increased significantly after modeling (*p* < 0.0001). (**C**) Change curve of fasting blood glucose of mice in each group during the bacterial transplantation process; the FDM group had the most remarkable decrease in blood glucose. (**D**) Insulin resistance index value, HOMA-IR = FBG*FINS/22.5, FBG (Fasting blood glucose), FINS (fasting insulin). (**E**) Levels of hemoglobin A1c (HbA1c) after intervention. The FDM, BIO and FMT groups showed different degrees of reduction, and the difference was statistically significant (*p* < 0.05). high fat diet, 60% Kcal; i.p. STZ, intraperitoneal injection of streptozotocin; HFD-STZ, type 2 diabetes (T2D) group, gavaged with PBS; FDM, fecal dominant microbiota group, the group which received *Lactobacillus* transplantation; BIO, the group which received *Bifidobacterium* transplantation; FMT, fecal microbiota transplantation group, gavaged with fecal supernatant; control, 4.9% fat diet group, gavaged with PBS. The number of mice in each group at the end of the experiment was as follows: control (*n* = 10), HFD-STZ (*n* = 4), FDM (*n* = 5), BIO (*n* = 5), and FMT (*n* = 6). * (*p* < 0.05), **** (*p* < 0.0001), and ns (*p* > 0.05) represents the one-way ANOVA analysis with each group, # (*p* < 0.05), ### (*p* < 0.001), and #### (*p* < 0.0001) represents the one-way ANOVA analysis with the HFD-STZ group.

HFD/STZ allowed for the successful creation of a T2D model in mice (Figure 2A), with a fasting blood glucose (16.27 ± 0.99 mmol/L, *p* < 0.0001) significantly higher than that of the control group (4.04 ± 0.25 mmol/L) (Figure 2B). Transplantation with different microbiota types was performed in each group. Due to cases of hyperglycemia syndrome in the early stage of treatment and experimental operation damage, the number of mice in each group was reduced. As treatment progressed, mouse death from hyperglycemia gradually decreased, the activity of the mice increased, the humidity of the bedding decreased, urine output significantly decreased, and overall survival status increased. After microbiota transplantation to treat the T2D mice, the FDM and BIO groups did not die as much in the late period as they did in the earlier period. 

To evaluate the effect of bacterial transplantation on the treatment of T2D, fasting blood glucose levels were measured after overnight fasting. Blood glucose levels decreased slowly as the treatment progressed (Figure 2C). The homeostasis model assessment of insulin resistance (HOMA-IR) showed a statistically significant decrease in the FDM and BIO groups, compared to the HFD-STZ group (*p* < 0.05) (Figure 2D). HbA1c levels, a long-term indicator of blood glucose levels, decreased in the FDM (8.1 ± 0.61%, *p* < 0.001), BIO (8.62 ± 0.54%, *p* < 0.05), and FMT (7.83 ± 0.71%, *p* < 0.001) groups compared with those in the HFD-STZ group (9.63 ± 0.85%) by the 12th week, and the difference was significant (Figure 2E). After 6 weeks of microbiota transplantation for T2D, both blood glucose and HbA1c were reduced, but they had not returned to a healthy state. The FDM group had the most significant effect on lowering blood glucose and HbA1c.

### 3.3. Bacterial Transplantation Ameliorates T2D-Induced Inflammation

**Figure 3:**
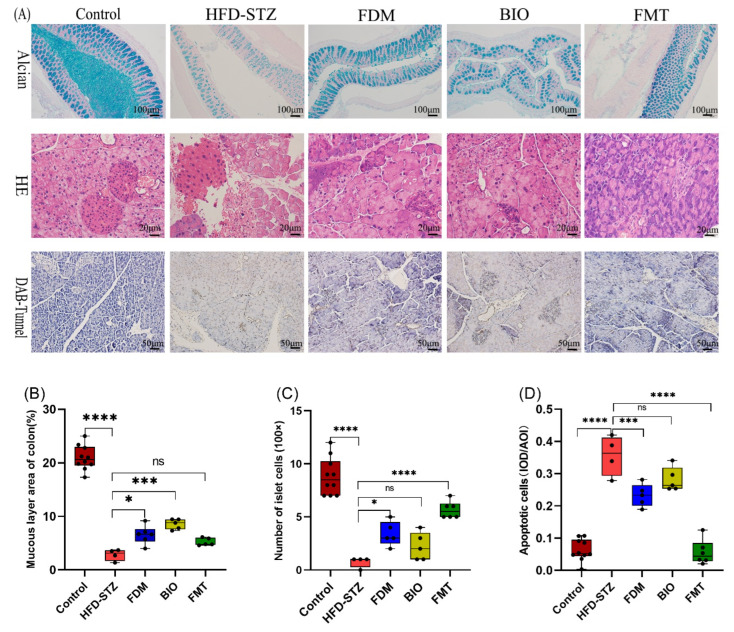
Effects of bacterial transplantation on colon and pancreas tissue. (**A**) Colon Alcian blue staining (100×), pancreas hematoxylin and eosin (HE) staining (400×), and pancreas TUNEL staining (200×). (**B**) The percentage area of the blue mucus layer of the colon in Alcian blue staining. (**C**) Calculated the average islet cells number per HE-stained pancreatic section in a microscope 100× field of view. (**D**) The IOD/AOI ratio of islet apoptotic cells. The number of mice in each group at the end of the experiment was as follows: control (*n* = 10), HFD-STZ (*n* = 4), FDM (*n* = 5), BIO (*n* = 5), and FMT (*n* = 6). * (*p* < 0.05), *** (*p* < 0.001), **** (*p* < 0.0001), and ns (*p* > 0.05) represents the one-way ANOVA analysis with each group.

Intestinal integrity plays an important role in maintaining the intestinal microecological balance. The mucus layer prevents bacteria from penetrating the intestinal barrier and entering circulation, while simultaneously protecting the intestinal acid-base balance. Alcian blue staining of colon tissue revealed differences in the mucus layer in the intestinal lumen of mice between groups [49]. There was a thick blue mucus layer in the control group, and the goblet cells were large, arranged normally, and numerous. In the HFD-STZ group, the mucus layer disappeared; it was incomplete, the intestinal villi became short and incomplete, and the folds became shallow (*p* < 0.0001). After treatment, the thickness of the colonic mucus layer increased to varying degrees, and the number of goblet cells increased. The number of goblet cells increased and the mucus layer thickened in the FDM group (*p* < 0.05) (Figure 3A,B), indicating that the strains in the FDM group changed the composition of intestinal microbiota, improved the dysbiosis, and played a role in promoting the regeneration of the intestinal mucus layer.

**Figure 4:**
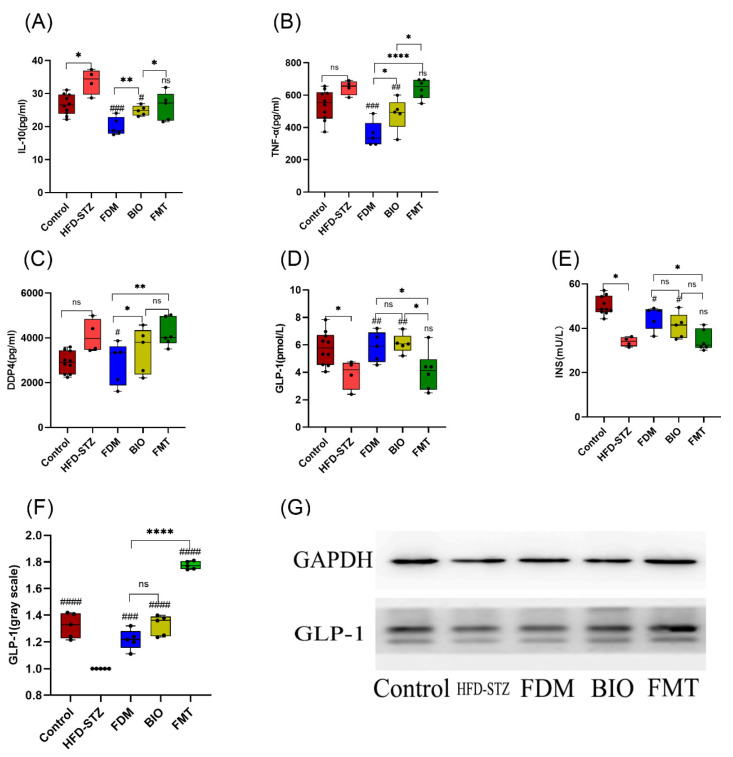
Effects of bacterial transplantation on levels of inflammatory factors. (**A**,**B**) Serum IL-10, and TNF-α levels in each group at 12th; the FDM group has the lowest levels of anti-inflammatory factor IL-10 and pro-inflammatory factors TNF-α. (**C**–**E**) Serum Dipeptidyl Peptidase-4 (DDP-4), glucagon-like peptide-1 (GLP-1) and insulin (INS) levels in each group at 12th; the FDM group has the highest levels of GLP-1 and INS. (**F**) Colonic GLP-1 western blotting gray value. (**G**) Western blotting of colonic GLP-1, which was secreted at the highest level in the FMT group. The number of mice in each group at the end of the experiment was as follows: control (*n* = 10), HFD-STZ (*n* = 4), FDM (*n* = 5), BIO (*n* = 5), and FMT (*n* = 6). * (*p* < 0.05), ** (*p* < 0.01), **** (*p* < 0.0001), and ns (*p* > 0.05) represents the one-way ANOVA analysis with each group, # (*p* < 0.05), ## (*p* < 0.01), ### (*p* < 0.001), and #### (*p* < 0.0001) represents the one-way ANOVA analysis with the HFD-STZ group.

Intestinal integrity is beneficial for reducing the release of inflammatory factors into the bloodstream. To understand the influence of the intestinal mucosal system on inflammation, serological inflammatory indicators were tested [50]. After transplantation of bacteria, TNF-α levels decreased significantly in the FDM (356.41 ± 78.6 pg/mL, *p* < 0.001) and BIO (483.24 ± 99.53 pg/mL, *p* < 0.01) groups to levels even lower than those seen in the control group (538.97 ± 93.9 pg/mL). No significant change was seen in the FMT group. However, the anti-inflammatory factor IL-10 level was significantly reduced in the FDM group (18.68 ± 3.31 pg/mL, *p* < 0.001) (Figure 4A,B). In general, the improvement in inflammation and decrease in levels of serum inflammatory factors were most clearly observed in the FDM group.

The pancreas is the most vital organ for the regulation of blood glucose levels. Together, inflammation and hyperglycemia syndrome lead to pancreatic β-cell damage. HE staining was used to observe the morphology, and TUNEL staining was used to observe apoptosis in pancreatic islet cells. In the control group, the islet cells were large, with round or elliptical cell clusters scattered among the acinar cells with clear boundaries. Regular cone-shaped or apoptotic cells were rare. Pancreatic islet cell clusters were rare in the HFD-STZ group, with brown-yellow apoptotic cells. In the three groups that received bacterial transplantation treatment, the number of pancreatic islet cell clusters increased, and the number of apoptotic cells decreased. After different treatments in the three groups, all showed a certain degree of islet regeneration. Surprisingly, in the FMT group, the number of islet cells was the largest and the apoptotic cells were the least (Figure 3A,C,D).

To further study the effect of intestinal microbiota on IR through improvement of inflammation, serum INS, GLP-1, and DDP-4 levels were tested. GLP-1 increased (5.84 ± 1.1 pmol/L, *p* < 0.05) and DDP-4 decreased (2861.77 ± 943.72 pg/mL, *p* < 0.05) in FDM group compared to the HFD-STZ group. In the FDM group, INS levels (47.71 ± 7.29 mU/L, *p* < 0.05) increased to the normal level (47.95 ± 7.61 mU/L) (Figure 4C–E). To further evaluate the level of intestinal hypoglycemic hormone, colon tissue was subjected to GLP-1 semi-quantitative western blotting protein analysis. In the FMT group, western blotting of the colon showed higher levels of GLP-1 (*p* < 0.0001) and lower levels of serum GLP-1, and this difference may reveal differences in the sites of hypoglycemic hormone by different microorganisms (Figure 4F–G).

### 3.4. Bacterial Transplantation Ameliorates T2D-Induced Fatty Liver

**Figure 5:**
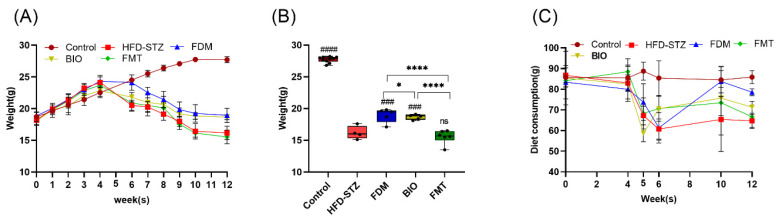
Effects of bacterial transplantation on weight and diet. (**A**) Body weight change curve during the intervention process. (**B**) Mice weight after intervention, in which the FDM group increased significantly (*p* < 0.001). (**C**) Change curve of the diet consumption of 5 mice/week in the intervention process, which was restored to a relatively constant level from a diet with lower quantities of food following modeling. The number of mice in each group at the end of the experiment was as follows: control (*n* = 10), HFD-STZ (*n* = 4), FDM (*n* = 5), BIO (*n* = 5), and FMT (*n* = 6). * (*p* < 0.05), and **** (*p* < 0.0001) represents the one-way ANOVA analysis with the each group, # (*p* < 0.05), ### (*p* < 0.001), and #### (*p* < 0.0001) represents the one-way ANOVA analysis with the HFD-STZ group.

To study the effect of microbiota transplantation on lipid metabolism, the weight and diet of mice was monitored. The hyperglycemia after modeling caused the weight to drop sharply to 20.53 ± 0.79 g. At the end of the treatment, the FDM (18.94 ± 1.11 g, *p* < 0.001) and BIO (18.66 ± 0.4 g, *p* < 0.001) groups increased more than HFD-STZ (16.18 ± 1.04 g) (Figure 5A,B). The average amount of food consumption per 5 mice per week was determined by modeling (60.75 ± 4.92 g) and stabilized to a relatively constant level (78.56 ± 1.72 g, *p* < 0.001) at the 12th week (Figure 5C). As the colonic mucus layer and appetite loss of the T2D mice were restored to a certain extent after the microbiota transplantation, the weight of mice was maintained at a certain level in the later period and no longer decreased. Among them, the FDM group exhibited the greatest recovery, while the FMT group showed the least amount of recovery.

**Figure 6:**
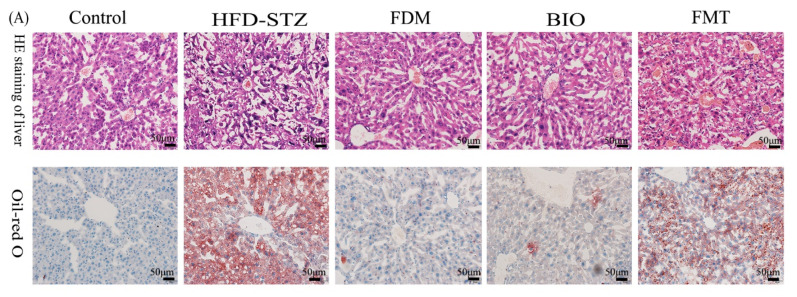

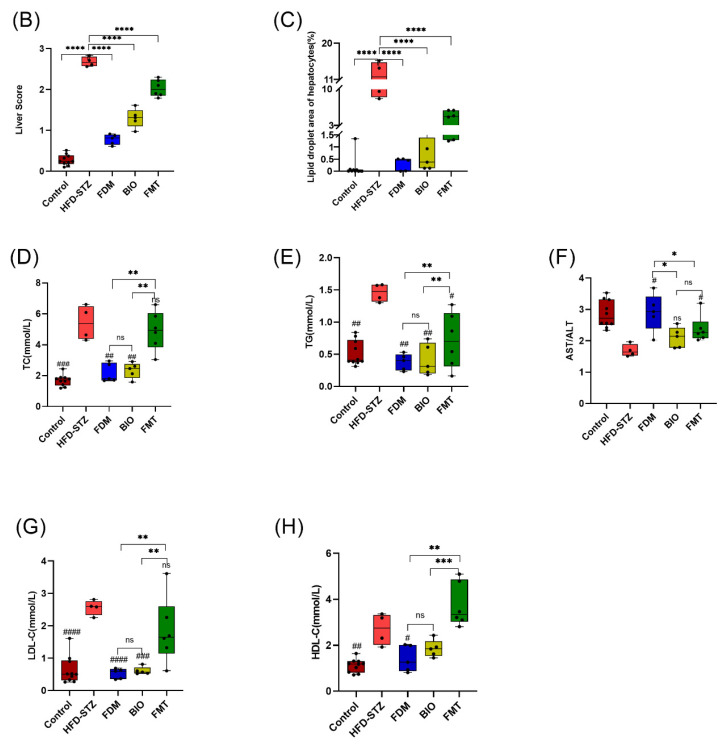
Effects of bacterial transplantation on fatty liver and blood lipids. (**A**–**C**) Images of liver tissues stained with hematoxylin and eosin (HE; 200×) and oil red O (200×). There are obvious pseudo-lobules and lipid droplets in the HFD-STZ group. The three groups receiving bacterial transplantation treatment were significantly transformed to normal liver cell tissue, of which the FDM group recovered the most notably (*p* < 0.0, 001). (**D**–**H**) Plasma total cholesterol (TC), triglycerides (TG), aspartate aminotransferase/alanine aminotransferase (AST/ALT), low-density lipoprotein cholesterol (LDL-C), and high-density lipoprotein cholesterol (HDL-C) levels. The serum levels of TC, TG, and LDL-C in the FDM and BIO groups decreased significantly, and the HDL-C increased significantly in the FMT group. The number of mice in each group at the end of the experiment was as follows: control (*n* = 10), HFD-STZ (*n* = 4), FDM (*n* = 5), BIO (*n* = 5), and FMT (*n* = 6). * (*p* < 0.05), ** (*p* < 0.01), *** (*p* < 0.001), **** (*p* < 0.0001), ns (*p* > 0.05) represents the one-way ANOVA analysis with the each group, # (*p* < 0.05), ## (*p* < 0.01), ### (*p* < 0.001), and #### (*p* < 0.0001) represents the one-way ANOVA analysis with the HFD-STZ group.

To understand the impact of microbiota transplantation on liver lipid metabolism, HE staining of liver tissues was performed. It was revealed that the structure of liver cells was generally normalized and repaired to a certain extent, structure of hepatic lobule became normalized, and size of the pseudo-lobule was reduced in the three groups in which microbiota transplantation was performed. Oil red O staining further showed that, in these groups, the degree of hepatic steatosis was reduced, and the hepatic lipid droplets were significantly reduced. The FDM group recovered the most (*p* < 0.0001) (Figure 6A–C). In serology, the effect of bacterial transplantation on hepatic steatosis was also obvious. Compared with that in the HFD-STZ group (5.26 ± 1.46 mmol/L), TC level was significantly reduced in the FDM (2.21 ± 0.68 mmol/L, *p* < 0.01) and BIO (2.24 ± 0.54 mmol/L, *p* < 0.01) groups. Compared with the HFD-STZ (1.41±0.22 mmol/L), TG levels in the FDM (0.4 ± 0.15 mmol/L, *p* < 0.01) and BIO (0.43 ± 0.27 mmol/L, *p* < 0.01) groups recovered to normal levels (0.57 ± 0.27 mmol/L). Compared with HFD-STZ group (2.56 ± 0.23 2.56±0.23), LDL-C levels also significantly decreased in FDM (0.53 ± 0.16 mmol/L, *p* < 0.0001) and BIO (0.61 ± 0.12 mmol/L, *p* < 0.001) groups. AST/ALT ratio increased to 3.11 ± 1.52 (*p* < 0.05) in FDM group, but the increase in HDL-C level was not obvious. In the FMT group, TC (5.08 ± 2.01 mmol/L), TG (0.81 ± 0.62 mmol/L), and LDL-C (1.85 ± 1.02 mmol/L) levels and AST/ALT ratio (2.96±1.18) did not decrease to the extend seen in the other groups. However, HDL-C (3.56 ± 1.12 mmol/L) levels showed a significant increase compared with those in the control group (1.09 ± 0.33 mmol/L) (Figure 6D–H). Overall, microbiota transplantation markedly improved lipid degeneration, and the FDM group had the best therapeutic effect.

## 4. Discussion

Probiotics have a variety of functions, such as antioxidant, anti-cancer, anti-inflammatory, improved metabolism, and immune function [51,52]. A growing number of studies have shown that probiotics have great potential in treating metabolic diseases, such as diabetes and obesity [53,54]. *Lb.* and *B. longum.*, bacteria present in the intestine, are beneficial to health and recommended to be added to one’s daily diet by the International Scientific Association for Probiotics and Prebiotics [55,56,57,58]. *Lb.* metabolites are closely related to glucose and lipid metabolism and work synergistically to work against obesity, non-alcoholic fatty liver, IR, and T2D [58]. However, the therapeutic effects of these probiotics on T2D remain controversial. In this experiment, a new strain of *Lb.* CGMCC 21661 was screened, and it was verified that it could reduce blood glucose and lipids in T2D mice through bacterial transplantation. In this experiment, levels of blood glucose, an excellent indicator of diabetes, improved significantly following bacterial transplantation. HbA1c is the product of the hemoglobin glycation reaction and can effectively reflect the average blood glucose level over 8–12 weeks [59]. Detection of HbA1c helps accurately understand the long-term effects of various bacterial groups on diabetes. *Lb.* CGMCC 21661, *B. longum. Subsp.*, and FMT all had hypoglycemic effects to varying degrees. *Lb.* CGMCC 21661 had the most obvious direct hypoglycemic effect. HbA1c, which reflects the long-term blood glucose index, also showed a decrease. 

Related studies have shown a close relationship between intestinal microbes, inflammation, and pancreatic damage during T2D [60]. Inflammatory factors (TNF-α, and IL-10), oxidative stress, and macrophage infiltration markers are positively correlated [61]. In the intestinal microbiota, endotoxins, such as LPS, trigger endotoxemia, which can cause inflammatory damage to multiple organs. Endotoxemia has been proven to be an important factor in the development of IR, and non-alcoholic fatty liver [60]. Pro-inflammatory factors (such as TNF-α) can induce serine phosphorylation of insulin receptor substrate and convert it into an inhibitor of insulin receptor tyrosine kinase activity in vitro, increasing IR [62,63]. Short-chain fatty acids activate G-protein coupled receptor GPR43 on intestinal L cells, increasing the secretion of secretin GLP-1 and peptide YY, and reducing IR. Probiotics can adhere to the surface of the intestinal mucosa through adhesins [64] and covalently bind to the intestinal mucosal cell receptor protein to form a biological barrier. Probiotics repair the intestinal barrier and prevent bacterial and inflammatory factors from entering the blood from the intestine. LPS activates the inflammatory pathway of pathogen-related molecular patterns through nuclear factor κB, which is blocked by probiotics [65]. This inhibition of inflammatory pathways prevents the occurrence of inflammatory reaction and reduces islet cell damage caused by inflammation. *Lb.* showed higher anti-inflammatory effects and provided greater protection against inflammatory reaction than *B. longum. subsp.* And fecal microbiota on transplantation. The levels of the pro-inflammatory factor TNF-α were significantly reduced following transplantation with lactobacilli. Colon Alcian staining showed that the intestinal mucosa in the FDM group was thickened and cup-shaped. Cell recovery was obvious, and the integrity of the intestinal barrier was restored to a large extent by treatment with *Lb.*, suggesting this bacterium helps maintain the balance of the intestinal microecosystem. The increase and decrease in GLP-1 and DDP-4 levels after *Lb.* CGMCC 21661 transplantation once again confirmed that improving the intestinal micro-ecosystem is linked to the production and secretion of secretin. It further confirmed that islet cell histology improved, and IR decreased following transplantation. 

In this study, following bacterial transplantation, mouse weight remained in a stable low-weight state, but not concerningly low, and the blood lipid index was reduced. This indicates that bacterial transplantation, particularly with *Lb.* CGMCC 21661, had a significant effect on obesity and lipid metabolism. T2D is related to obesity and IR caused by local fat accumulation. Excessive fat accumulation in the liver, pancreas, and other tissues aggravates IR and causes T2D [66]. The intestinal microbial metabolite acetic acid is a precursor for inducing fatty acid desaturation, which promotes fatty acid oxidation and reduces weight [65]. Butyrate, another microbial metabolite, is an energy source for colon cells and functions together with propionic acid to improve glucose homeostasis. Microbial metabolites induce antilipolytic activity, activate GPR43 in white adipose tissue, inhibit fat accumulation in adipose tissue, and promote glucose and lipid metabolism in other tissues [66]. As one of the most important probiotics, *Lb.* produces conjugated linoleic acid (c9, t11-CLA), and its intermediate product activates peroxisome proliferator-activated receptor gamma, which increases adiponectin production and glucose uptake [67,68]. Concurrently, the decomposition of short-chain fatty acids by *Lb.* induces PPARγ to increase the expression of mitochondrial uncoupling protein 2 and increase the ratio of AMP to ATP, reduce obesity induced by an HFD, and reduce host TG involvement [67,69]. *Lb.* decomposes saturated long-chain fatty acids to regulate the intestinal microecological balance and stabilize the intestinal barrier while promoting microbial growth, which helps to reduce liver damage caused by alcoholic fatty liver [68]. Moreover, *Lb.* can reduce the plasma and liver TG and LDL-C levels caused by an HFD, thereby ameliorating the imbalance of microbiota and increasing insulin sensitivity by regulating lipid metabolism [70,71]. The experiments described in this paper showed that, following bacterial transplantation, mice weight reduced to a stable low-weight state and the blood lipid index was reduced. This indicates that bacterial transplantation, particularly with *Lb.* CGMCC 21661, had a significant effect on obesity and lipid metabolism. The detection of islet cells indicates that *Lb.* CGMCC 21661 can improve lipid metabolism and that inflammatory reaction jointly regulates insulin sensitivity.

## 5. Conclusions

In recent years, increasing evidence suggests that the gut microbiota plays an important role in the development of metabolic diseases. In this study, an HFD/STZ was used to establish T2D related to IR in mice, and the glucose- and lipid-lowering effects of different microbiota transplantation programs were investigated. We found that *Lb.* CGMCC 21661 was superior to *B. longum. subsp.* and FMT in reducing blood glucose and repairing tissue damage in the liver, pancreas and colon. *Lb.* CGMCC 21661 has obvious advantages in reducing blood glucose levels by reducing inflammatory reaction and improving lipid metabolism. Mice with severe diabetes had only moderate diabetes following these experiments, and the improvement in their condition was long term. In future, experiments should focus on the mechanism through which *Lb.* CGMCC 21661 allows these improvements to occur. Surprisingly, the lipid-lowering effect of *Lb.* CGMCC 21661 was also found to reduce insulin resistance, thus providing new hope for the treatment of obesity and fatty liver disease. It is also possible that, combined with other probiotics that help repair pancreatic islet tissue, *Lb.* CGMCC 21661 could become a new treatment for T2D.

## Figures and Tables

**Table 1:** Annotation of virulence factors of pathogenic bacteria (VFDB) database.

Dataset	Total Proteins	Predicted VFDB Proteins
setA	3730	216
setB	3730	227

**Table 2:** Functional annotation of the comprehensive antibiotic resistance database (CARD).

Total Proteins	Predicted Antibiotic Resistance Proteins	Ratio (%)
3730	51	1.37

**Table 3:** Annotation of pathogen-host interaction (PHI).

Total_Proteins	Predicted_PHI	Ratio (%)
3730	92	2.47

## Data Availability

The original contributions presented in the study are included in the article/Appendix A, further inquiries can be directed to the corresponding author/s.

## Appendix A

**Table A1:** Normal diets—Fat: 4.9%.

Control diets	Grams/g
water	100
Protein	180
Fat	40
Fiber	50
Crude ash	80
Ca^2+^	10
P^5+^	6
Ca^2+^/P^5+^	1.2/1
Mineral	9.2159
Amino acid	76.6
Vitamin	1.34312
Total	473.15902

Note: Feed content per kilogram.

16S rRNA is a sequence encoding rRNA on bacteria. It is present in all bacterial genes. It is highly conservative and specific. It is used for strain identification. The identification result is *Lactobacillus.*
